# Building Blocks of Temporal Filters in Retinal Synapses

**DOI:** 10.1371/journal.pbio.1001973

**Published:** 2014-10-21

**Authors:** Bongsoo Suh, Stephen A. Baccus

**Affiliations:** 1 Department of Electrical Engineering, Stanford University, Stanford, California, United States of America; 2 Department of Neurobiology, Stanford University School of Medicine, Stanford, California, United States of America

## Abstract

Sensory systems must be able to extract features of a stimulus to detect and represent properties of the world. Because sensory signals are constantly changing, a critical aspect of this transformation relates to the timing of signals and the ability to filter those signals to select dynamic properties, such as visual motion. At first assessment, one might think that the primary biophysical properties that construct a temporal filter would be dynamic mechanisms such as molecular concentration or membrane electrical properties. However, in the current issue of *PLOS Biology*, Baden et al. identify a mechanism of temporal filtering in the zebrafish and goldfish retina that is not dynamic but is in fact a structural building block—the physical size of a synapse itself. The authors observe that small, bipolar cell synaptic terminals are fast and highly adaptive, whereas large ones are slower and adapt less. Using a computational model, they conclude that the volume of the synaptic terminal influences the calcium concentration and the number of available vesicles. These results indicate that the size of the presynaptic terminal is an independent control for the dynamics of a synapse and may reveal aspects of synaptic function that can be inferred from anatomical structure.

## Abstract 2

We discuss recent findings that uncover how the physical size of synaptic terminals contributes to the temporal filtering of retinal synapses.

## Introduction

Sensory signals are composed of a combination of steady and rapidly changing features: for instance, moving objects that traverse a steady background, tactile stimuli composed of steady pressure and fast vibrations, and musical notes with constant frequency that vary in loudness. Neurons and synapses represent these features using electrical and chemical signals that vary in time. In doing so, the internal timing that represents an external signal changes in order to perform computations such as detecting, discriminating, predicting, and acting upon properties of the world.

The vertebrate retina has served as a key system to discover how biophysical mechanisms of the nervous system perform computations. For functions such as encoding the direction of motion and detecting objects against a moving background, the temporal processing of signals is critical [1]–[5]. Much attention has been paid to the effect of molecular mechanisms on timing, such as ion channels, receptors, and molecules that control synaptic release [6],[7]. But a second class of mechanisms is the structure of the nervous system itself: axons that impose conduction delays, and the combined effects of neuronal morphology and electrical properties (e.g., resistance and capacitance) can influence the timing of membrane potential changes [8]. In this issue of *PLOS Biology*, Baden et al. show that temporal processing at a synapse can be controlled by the anatomical structure of the synaptic terminal, through its impact on the calcium signal that drives neurotransmission and on the number of available vesicles. Thus, the structure of a synapse does not simply act to bring two neurons in contact with each other; the volume of the presynaptic terminal can influence timing at the synapse.

A key advance of this paper is in understanding the mechanistic and quantitative relationship between synaptic structure and signal processing. This work highlights physical space as a limited resource and raises questions of how the size of a synapse is optimized. Furthermore, it raises the possibility that anatomical techniques can be used to infer the dynamic functional properties of synapses.

## How do Different Temporal Filters Operate?

In order to represent and discriminate different sensory features, many neurons are more sensitive to certain temporal patterns than others—a process known as temporal filtering. This filtering process has a critical effect on how action potentials represent information—the neural code. Thus, determining the mechanisms of how different temporal filters are implemented is crucial for understanding how the brain represents the external world.

The essence of a filter—whether one for water or for electrical signals—is that it allows certain things to pass while rejecting others. More generally, a filter applies a weighting to different types of objects or signals, so that some pass freely, some are attenuated, and others are reversed in sign. Filters can be used to emphasize a range of input, such as high acoustic frequency, or special patterns, like an individual's voice or even a particular word. A visual spatial filter may reject fine textures but transmit uniform regions of intensity. Similarly, a temporal filter applies a different weighting to different signals as a function of time delay, so that recent inputs receive a different weighting than signals further in the past. Thus, temporal filtering is pervasive in the nervous system to extract and represent features that are relevant for specific behaviors.

**Figure 1:**
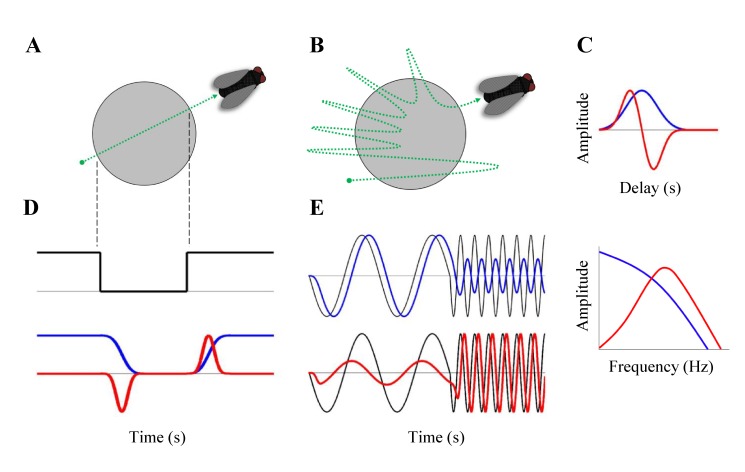
Effects of different temporal filters. **A**, A fly moves over a receptive field of a cell (gray circle) that depolarizes in response to light. The dotted green line shows the trajectory of the fly moving from bottom left to upper right at a constant velocity. **B**, A fly flying over the receptive field with changing acceleration; movement in and out the receptive field starts slowly, then becomes faster. **C**, Two example temporal filters of a cell are shown in the top panel—a monophasic (*Blue*) and a biphasic filter (*Red*), having a low-pass and a band-pass frequency response, respectively, as shown in the bottom panel. **D**, *Top*: The approximate change in light intensity over the receptive field elicited by the fly's trajectory in **A** is shown. *Bottom*: The light intensity generates different responses to the two different temporal filters from **C**. **E**, The approximate pattern of light intensity generated by the fly's movement in **B**, having slow and fast frequency components, is passed through the two filters from **C**. *Top*: The output of the low pass filter (*blue*) is compared to the light input (*black*). *Bottom*: The output of the band pass filter (*red*) is compared with the light input (*black*).

As an illustration of the effects of different temporal filters, consider when a fly moves across the receptive field of a cell with a constant velocity, causing the light intensity averaged over the receptive field to drop, remain constant, and then increase (Figure 1A). Figure 1C illustrates two different types of filters that a cell might have: either a monophasic (having one positive or negative phase) or biphasic filter (having both positive and negative phases). One can think of these temporal filters as the average response to a brief flash of light, i.e., a photon. If one were to consider a simplified (linear) model of the cell, in which the effects of all photons were the same and those effects would simply sum, then the cell's temporal filter alone would enable the prediction of responses to other stimuli. Figure 1D shows that in response to the constant velocity stimulus (Figure 1A), the cell with a monophasic filter follows the trajectory of a stimulus with some delay and extracts slow components of the stimulus. In contrast, a biphasic filter captures sharp transitions of a stimulus but responds little to the constant value. For a more complex trajectory with fast and slow frequency components (Figure 1B), the monophasic filter acts as an integrator or a low-pass filter, conveying slower components more effectively; the biphasic filter operates more like a differentiator or a band-pass filter, which signals higher frequency components but attenuates both the highest and lowest frequencies (Figure 1E). Thus, different filters extract different properties of the signal.

## Shaping Visual Signals through Retinal Circuitry

**Figure 2:**
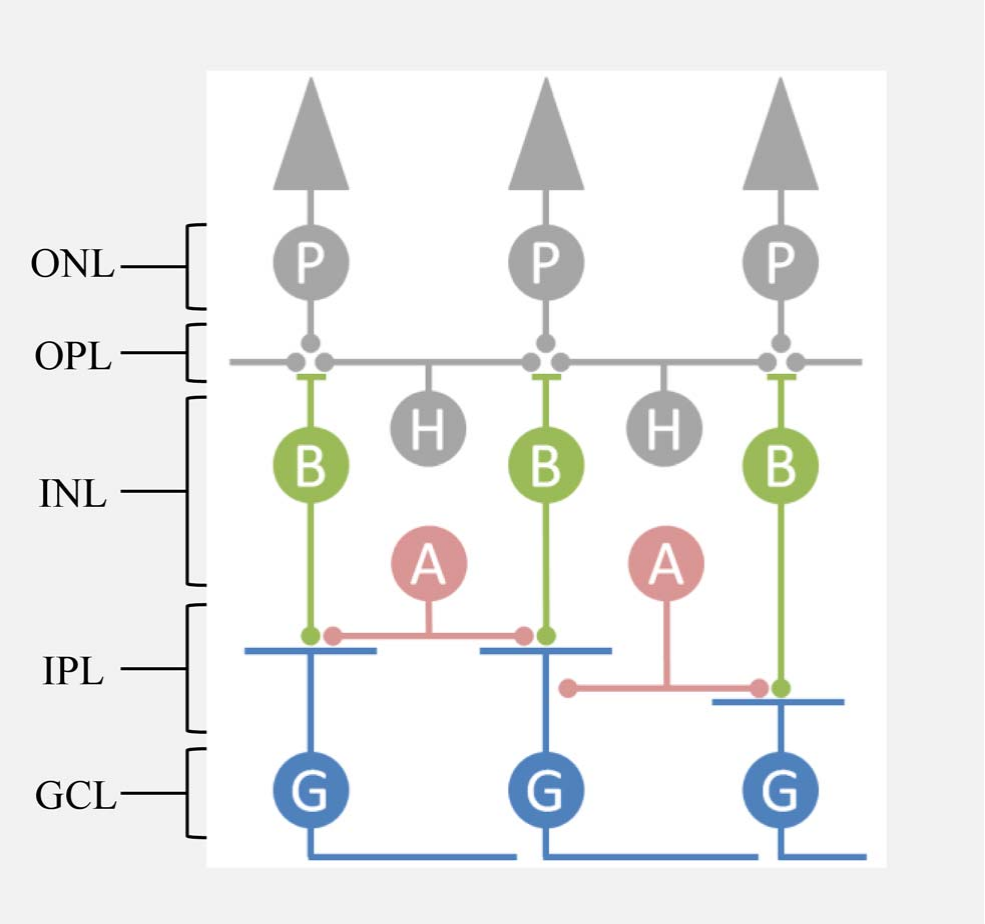
Organization of the retina. A schematic diagram of the vertebrate retina is shown. Light intensity is transformed into electrical signals by photoreceptors (P) in the outer retina. Bipolar cells (B) deliver signals from photoreceptors to ganglion cells (G). Inhibitory horizontal cells (H) and amacrine cells (A) further transform signals, in complex ways not discussed here. Different bipolar cells synapse onto different stratification layers in the IPL. ONL, Outer nuclear layer; OPL, Outer plexiform layer; INL, Inner nuclear layer; IPL, Inner plexiform layer; GCL, Ganglion cell layer.

The retina converts visual signals captured by photoreceptors into a sequence of action potentials generated by more than 20 types of ganglion cells, the output neurons of the retina, which differ in their temporal filtering and their preferred visual features (Figure 2). About ten types of bipolar cells bridge between photoreceptors and ganglion cells, whereas inhibitory horizontal cells and amacrine cells implement added processing to the input and output of the bipolar cells, respectively [9]. The most complex connectivity and computations are found within the inner plexiform layer (IPL), where more than 30 classes of inhibitory amacrine cells generate nonlinear transformations of visual signals such as producing selectivity for the direction of motion and sensitivity to object motion [10]–[13]. Bipolar cells differ in dendritic field size, receptor types and the projection depth of axon terminal stratification within the IPL [14]–[16]. Based on studies in the mammalian retina, the stratification of bipolar cells is arranged roughly according to similar temporal response properties. The most familiar segregation of bipolar cell terminals in different sublamina is that of ON bipolar cells—that depolarize with increasing light intensity—from OFF bipolar cells. In addition, bipolar cells terminating in the intermediate levels of the IPL tend to act more like a band-pass filter [9],[17]–[19].

## Mechanisms Contributing to Filtering at Bipolar Cell Synaptic Terminals

**Figure 3:**
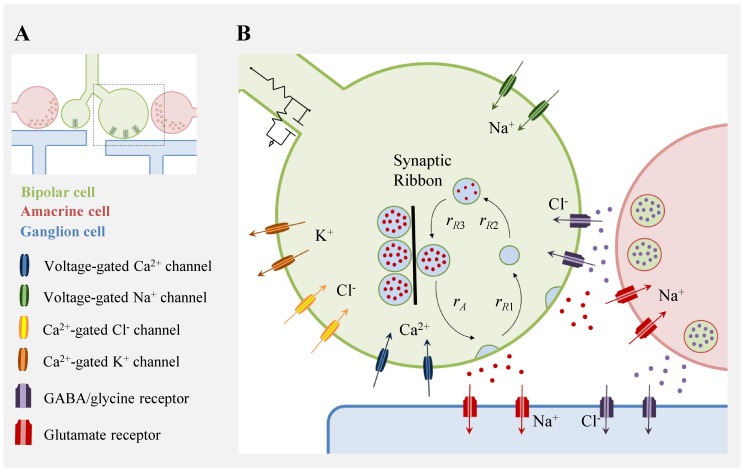
Mechanisms of temporal filtering at the bipolar cell terminal. **A**, A schematic diagram is shown of a bipolar cell axon with large and small synaptic terminals, synapsing on amacrine and ganglion cells. **B**, A close-up view of the large terminal (dashed square in **A**) shows some mechanisms that influence temporal filtering. When the electrical signal enters the terminal, it is transformed by the low-pass filter, shown as the circuit diagram, formed by axial resistance, membrane resistance, and capacitance. In some bipolar cells, voltage-gated sodium channels generate spikes at the terminal [28]. Depolarization opens voltage-gated calcium channels, and the influx of calcium ions activates vesicle fusion and neurotransmitter release, signaling to amacrine and ganglion cells through glutamate receptors. Vesicle cycling is described mathematically based on kinetic measurements by an activation rate constant (*r_A_*) and the recovery rates (*r_R_*_1_, *r_R_*_2_, *r_R_*_3_). The rate constant *r_R_*_1_ is related to the rate of endocytosis, and *r_R_*_2_ and *r_R_*_3_ correspond to the rate of refilling of two “pools” of vesicles known as the recycling pool and the readily releasable pool (RRP), respectively [25]. Intracellular calcium ions diffuse from calcium channels to calcium-gated potassium and chloride channels, which produce a delayed hyperpolarization, attenuating steady inputs and contributing to a biphasic band-pass filter. Finally, GABAergic or glycinergic inhibitory amacrine cells have pre- and post-synaptic control over signal transmission, also contributing to a biphasic filter.

It is difficult to ascertain how a property such as temporal filtering is generated because it is a result of a combination of many effects. Each transduction cascade, ion channel, membrane capacitance, and neurotransmitter receptor that carries the signal from photoreceptor to ganglion cell influences the timing of the response—there is no “one mechanism” of temporal filtering. Taking as an example the bipolar cell synaptic terminal, light-evoked depolarization activates voltage-gated calcium channels, triggering synaptic vesicle fusion and neurotransmitter release (Figure 3). Numerous mechanisms and factors involved in these operations contribute to temporal filtering of the signal. The same signal sent to different synaptic terminals undergoes different temporal filtering; the terminals have different cellular properties, including ion channels, size of vesicle pools, and membrane time-constants, that determine the frequency range of a low-pass filter.

Even at this one synapse, many different ion channels influence temporal filtering (Figure 3B). The kinetics of voltage-gated calcium channels greatly affect the temporal bandwidth of synaptic vesicle fusion; activation and inactivation dynamics and the conductance of the channel affect not only the calcium current [20] but also calcium spikes, which amplify synaptic release in short time intervals [17],[21],[22]. Calcium dynamics in turn influence vesicle fusion, neurotransmitter release, and the transition rate of vesicles between different synaptic vesicle pools [23],[24]. The transition rates influence the depletion of the readily releasable pool (RRP)—a mechanism that contributes to temporal filtering and adaptation [25]–[27]. The concentration of intracellular calcium ions also influences other ion channels such as calcium-gated chloride and potassium channels, and this process relies on the time-dependent diffusion of calcium ions [18]. Moreover, in some bipolar cells spiking responses produced by calcium currents in fish—or sodium currents in mammals—emphasize fast temporal components of the membrane potential [28]–[30]. In general, mechanisms that produce a delayed inhibition or decrease in the signal will tend to attenuate steady inputs, creating a band-pass filter.

Besides intrinsic mechanisms, interactions with inhibitory amacrine cells further influence temporal filtering. Approximately 30 types of amacrine cells modulate excitatory pathways, creating diverse temporal effects in ganglion cells [9]. For example, polyaxonal and starburst amacrine cells provide inhibition to object motion-sensitive and direction-selective ganglion cells, respectively [4]. In addition, amacrine cells release the inhibitory neurotransmitters of GABA or glycine, and depending on the type of receptors present, amacrine cells regulate temporal transmission differently—such as shaping the signal to be transient or sustained [31].

## Linking Temporal Filtering and Adaptation to Synaptic Terminal Volume

In the current issue of *PLOS Biology*, Baden et al. add a new mechanism to this list—the volume of the synaptic terminal—and analyze the contribution of this mechanism to temporal filtering. A key tool in uncovering their findings was a novel in vivo imaging approach in zebrafish. Using genetically encoded optical indicators targeted specifically to synapses—sypHy, which detects vesicle fusion, and SyGCaMP2, which detects presynaptic calcium—the authors observe that bipolar cell synaptic terminals with a smaller volume generate faster, larger, and more transient changes in intracellular calcium and vesicle release. Moreover, smaller terminals show greater adaptation to contrast, reducing their output over time during a higher contrast stimulus. This process of adaptation allows a cell to avoid saturation by strong stimuli, thus enabling it to use its dynamic range more efficiently [32].

The experimental evidence reveals a correlation of synaptic terminal size with properties of temporal filtering and adaptation. But is that what really causes the difference in response properties? Because of known effects of GABAergic input to bipolar cell terminals on temporal processing [33], the authors used pharmacology to rule out the possibility that differential GABAergic feedback might be responsible for differences in bipolar cell responses. However, the ideal experiment to rule out other mechanisms—causally manipulating the size of the terminal and nothing else—isn't feasible. Instead, the authors perform this manipulation in a biophysical computational model of the synaptic terminal, and they find that if all other variables are held constant, the size of the terminal alone is enough to explain the observed differences in response properties. Of course, there may still be other differences in biophysical mechanisms between the two classes of terminals, but these aren't necessary to explain the different responses. In this sense, computational models play an important role in testing hypotheses, especially for technically unrealistic experiments.

The model offers explanations as to how the smaller terminal size changes signal processing. The authors conclude that the faster, larger changes in intracellular calcium result from the higher surface to volume ratio of smaller terminals. With an equal conductance per membrane area, smaller terminals will experience a greater change in concentration that will equilibrate more quickly, due to diffusion across a smaller volume. The greater adaptation in smaller terminals is consistent with the notion that vesicle depletion is a primary source of contrast adaptation—smaller terminals may deplete more quickly because of a smaller reserve of vesicles.

Finally, the authors explain that the band-pass filtering of smaller terminals also derives from greater vesicle depletion. This effect occurs because small terminals with a smaller vesicle capacity produce a transient output in response to a steady input. Thus vesicle depletion has two effects: a nonlinear, time-dependent change in gain and an influence on the bandwidth of temporal filtering [25].

As mentioned in Baden et al., it will be important to determine which ganglion cell types receive input from large or small bipolar cell terminals. One cell type of particular interest is the primate parasol ganglion cell, which has a more transient response than the smaller “midget” ganglion cells. Parasol ganglion cells also adapt more strongly, a behavior that may be a functional benefit because they pool over more bipolar cells than do the smaller midget ganglion cells and thus may have a greater need to adapt [34],[35]. It will be of interest to determine how much these differences are due to the differing morphologies of the synaptic terminals of “diffuse” bipolar cells, which target parasol ganglion cells, versus midget bipolar cells, which target midget ganglion cells.

In addition to whether different synaptic terminals target different types of ganglion cells, it will be useful to understand whether different bipolar terminals receive different types of temporal inputs. Although it is possible that inputs to large and small terminals are the same, perhaps for different bipolar cells, the temporal filtering of the synapse is a “matched filter” to that of the soma; this matching might be the case if the terminal is optimized to detect voltage changes in the same range of that as the soma [36].

The authors show that smaller synaptic terminals have both larger bandwidth (a wider response range of temporal frequencies) and higher gain. This is surprising, as most signaling systems like electronic amplifiers and photoreceptors have a fundamental trade off: if amplification is greater, the temporal bandwidth is necessarily smaller. This constant factor is known as the gain-bandwidth product [37]. What then is lost by this added performance? Perhaps larger synaptic terminals have a larger dynamic range of signaling because they have a larger reserve of vesicles, and thus they need to adapt less. Similarly, it will be interesting to learn whether larger terminals can transmit with a higher signal-to-noise ratio given the larger number of vesicles. Indeed, an analogous phenomenon has been shown in the auditory system, that large terminals with large vesicle pools and large dynamic range showing less adaptation have a higher signal-to-noise ratio [38],[39].

## Conclusions

There have long been attempts at connecting neural structure to function [40]. Recent efforts to reconstruct every synaptic connection in a neural circuit—an approach known as connectomics—can reveal the presence of synaptic connections, but face the critical barrier of a lack of functional information about different synapses [41]. Although further study and validation are needed, the findings of Baden et al. raise the possibility that certain properties of synapses might be inferred directly from their structure.

The nervous system performs functions such as the discrimination of sensory stimuli using cells and synapses with limited resources, such as energy and time. The novel connection reported by Baden et al. between synaptic terminal volume, temporal filtering, and adaptation brings into focus the question of how biophysical mechanisms and structures are optimized to perform computations. Neural circuits use their resources of energy, physical space, and time to achieve the performance criteria of information transmission, amplification, filtering, and dynamic range. Strategies of adaptation allocate these resources dynamically based on the recent history of input [42]. It will be interesting to see which factors tradeoff with each other, and what principles of resource allocation, such as the maximization of information transmission or energy efficiency, influence neural mechanisms and structures.

## Abbreviations

IPL: Inner Plexiform Layer
RRP: Readily Releasable Pool
